# Community-Led Defaulter Tracking for Catch-Up Vaccination: Implementation Experience in Uganda, 2022 and 2024

**DOI:** 10.3390/vaccines14060490

**Published:** 2026-05-30

**Authors:** Joseph Magoola, Brooke N. Aksnes, Immaculate Ampeire, Yvette Wibabara, Ciara E. Sugerman, Kirsten Ward

**Affiliations:** 1 African Field Epidemiology Network, Lugogo House, Kampala 12874, Uganda; 2 Global Immunization Division, US Centers for Disease Control and Prevention, Atlanta, GA 30329, USA; 3 Expanded Program on Immunization, Ministry of Health, Kampala 7272, Uganda; 4 US Centers for Disease Control and Prevention, Kampala 1577, Uganda

**Keywords:** defaulter tracking, catchup vaccination, immunization, Uganda

## Abstract

Background: In Uganda, COVID-19-related disruptions increased the number of children who missed scheduled routine vaccination (defaulters). Identifying and following up with defaulter children is important for improving vaccination coverage. This paper describes Uganda’s experience in revitalizing community-led defaulter tracking to improve vaccination coverage post-COVID-19 in four purposefully selected districts. Methods: During two 6-month periods in 2022 and 2024, healthcare workers (HCWs) worked with village health teams (VHTs) to review health facility-based immunization registers, identify and track defaulters aged 0 to 59 months. VHTs visited identified defaulters’ homes, reviewed vaccination histories and reminded caregivers to bring defaulters to immunization sites for catch-up vaccination. Results: Overall, 20,922 defaulters were identified by health register review; VHTs located 15,749 (75.3%) through household visits, of whom 3688 (23.4%) were verified as previously vaccinated based on their home-based vaccination records, leaving 12,061 as true defaulters. Among the true defaulters, 9662 (80.1%) received at least one catch-up vaccination after follow-up by the VHT. The most frequently administered catch-up vaccines were measles–rubella first dose (MR1) at 55.4%, followed by diphtheria–tetanus–pertussis third dose (DTP3) at 48.3% and Bacillus Calmette–Guérin (BCG) at 47.4%. Among the 2399 children who remained unvaccinated after follow-up, the most common reasons were relocation outside the original catchment area (49.5%) and caregiver intention to vaccinate later (16.3%). Conclusion: Community-led defaulter tracking was feasible and improved vaccination uptake in post-COVID-19 Uganda. Strengthening the quality and availability of health facility immunization data, along with targeted community engagement, caregiver reminders and integrated vaccination services would improve identification and follow-up of defaulters, reducing population immunity gaps.

## 1. Introduction

Childhood immunization remains one of the most cost-effective public health strategies [1,2], substantially reducing morbidity and mortality worldwide [3,4,5]. Despite progress, sub-Saharan African countries still have millions of children considered zero-dose (received none of the recommended vaccines for their age, operationally defined as no record of the first dose of diphtheria–tetanus–pertussis-containing vaccine) [6], and under-immunized (received some, but not all recommended vaccines for their age) [7,8]. Large numbers of zero-dose and under-immunized children result in population immunity gaps leading to greater potential for sustained disease transmission and increased risk of outbreaks of vaccine-preventable diseases (VPDs). Understanding who is not immunized and where they are located is critical to address vaccination access and utilization gaps [9,10].

During the COVID-19 pandemic (2020–2021), routine vaccination coverage declined globally, with first-dose measles-containing vaccine (MCV1) coverage falling from 85% pre-pandemic in 2019 to 81% in 2021, and diphtheria–tetanus–pertussis-containing vaccine third-dose (DTP3) coverage decreasing from 86% in 2019 to 81% in 2021. In Africa, six of fifteen countries reported > 10% decline in routine vaccine doses in the second quarter of 2020 compared with early 2020 [11,12,13], driven by reduced access to routine vaccination services due to movement restrictions (including lockdowns and social distancing), inadequate health facility staffing, and fear of COVID-19 infection in the community [14]. The number of zero-dose children in Africa increased from 7.1 million in 2021 to 7.7 million in 2022 [15]. Across the African continent, diphtheria–tetanus–pertussis-containing vaccine first-dose (DTP1) coverage fell from 75% pre-pandemic in 2019 to 73% in 2020, while DTP3 dropped from 77% in 2019 to 72% by 2021, continuing at that level through 2023 [16]. Coverage of MCV1 in Africa also declined during the pandemic period and remained below the global target of 95%, reflecting persistent immunity gaps requiring targeted recovery efforts [17], though remained below the global target of 95%. These disruptions have had lasting effects, with ten African countries now accounting for over 80% of zero-dose children worldwide [18]. During lockdowns from February to April 2020, Uganda specifically saw vaccination coverage among children under one year of age for DTP1 drop from 92% in 2019 to 79% in 2020, and coverage of MCV1 drop from 83% in 2019 to 68% in 2020 [19]. Identifying these children missing routine vaccines and providing an opportunity for catch-up vaccination is essential to optimizing population protection against VPDs.

A “defaulter” is a term used to describe children who initiate but do not complete the nationally recommended vaccination schedule [7] for their age. In this instance a defaulter refers specially to children identified through routine health facility immunization registers who had initiated vaccination but failed to return for a scheduled subsequent dose within the operational follow-up period. The goal of defaulter tracking systems is to ensure that every child receives age-appropriate vaccination according to the national immunization schedule, even if they are late [7]. The defaulter tracking approach works when immunization defaulters are accurately identified and tracked, and their vaccination outcomes are recorded and used to determine future vaccination needs. Defaulter tracking is a core part of Africa’s Reaching Every District/Reaching Every Child (RED/REC) strategy, which aims to optimize vaccination uptake through five locally focused approaches: planning and management of resources, supportive supervision, community links with service delivery, monitoring and use of data for action, and re-establishment of outreach services [20]. It is primarily operationalized through community linkage and data-driven monitoring, where communities help identify children who miss routine vaccines and health teams use local data to trace, follow-up, and reintegrate them into routine immunization services.

In 2002, Uganda began implementing the Village Health Team (VHT) program to address population health needs, health inequities and improve access to health services [21]. The VHTs are volunteers drawn from and who work within their communities, outside of healthcare facilities [22]. They are locally elected and are assigned between 25 and 30 households to help support health service delivery [23]. For routine immunization, VHTs do not administer vaccines and have not generally been involved in the follow-up of defaulters due to systemic and operational constraints, including lack of resources, inadequate tools and competing priorities. Evidence from immunization program reviews in Uganda has shown that low VHT involvement for community and social mobilization is associated with stagnated immunization performance [24].

Although defaulter tracking is recommended within Uganda’s immunization guidelines, implementation has historically been inconsistent in routine practice, particularly at the community level, due to operational and resource constraints. Following COVID-19-related disruptions to immunization services, there was a programmatic need to identify children who had missed scheduled vaccinations and support their return to routine services.

In Uganda, defaulter tracking is recommended within immunization guidelines, but it is not consistently implemented as part of routine immunization delivery in health facilities or outreach sites. Following the COVID-19 health system disruptions in Uganda, defaulter tracking was implemented to close VPD immunity gaps in four selected districts. VHTs were mobilized to work with their local health facilities to identify, locate and track un- or under-vaccinated children during two 6-month periods in 2022 and 2024. The implementation periods reflected phased programmatic rollout aligned with immunization recovery support and operational feasibility in priority districts. This manuscript describes Uganda’s approach to community-led immunization defaulter tracking in selected districts and outcomes related to identification, follow-up, and catch-up vaccination.

## 2. Materials and Methods

### 2.1. Setting

In Uganda, immunization defaulter tracking was implemented in four districts—three in the central region (Bukomansimbi, Nakasongola and Masaka) and one in the western region (Hoima)—during two 6-month periods: June–November 2022 and April–September 2024. These two implementation periods were determined by the availability of funding and operational resources to support the activity, rather than to assess temporal changes between the periods.

The districts were selected using a combination of criterion-based sampling [25], and programmatic feasibility. The implementation districts were purposively selected in collaboration with the Ministry of Health and district health teams based on immunization performance gaps, evidence of missed vaccinations following COVID-19-related service disruptions, and operational feasibility. Intervention timing aligned with program implementation cycles and phased scale-up opportunities. National administrative vaccination coverage data from 2016 to 2020 was collated by year and district. Five-year averages were calculated for the following immunization performance indicators: DTP1 coverage, DTP3 coverage, DTP1-DTP3 dropout (i.e., proportion of individuals who received DTP1 but not DTP3) and number of un-immunized children. Districts were eligible for selection if they met two or more of the following criteria: DTP1 and DTP3 coverage < 80%, DTP3-DTP1 dropout rate > 10%, and proportion of unimmunized children > 10% based on five-year averages from 2016 to 2020. Based on these criteria, 44 of 135 districts were eligible for selection, and from these four were selected based on programmatic considerations, including districts with the highest need for immunization technical assistance as they had no external technical assistance for immunization at the time of selection.

### 2.2. Implementation of the Defaulter Tracking Approach in Uganda

**Figure 1:**
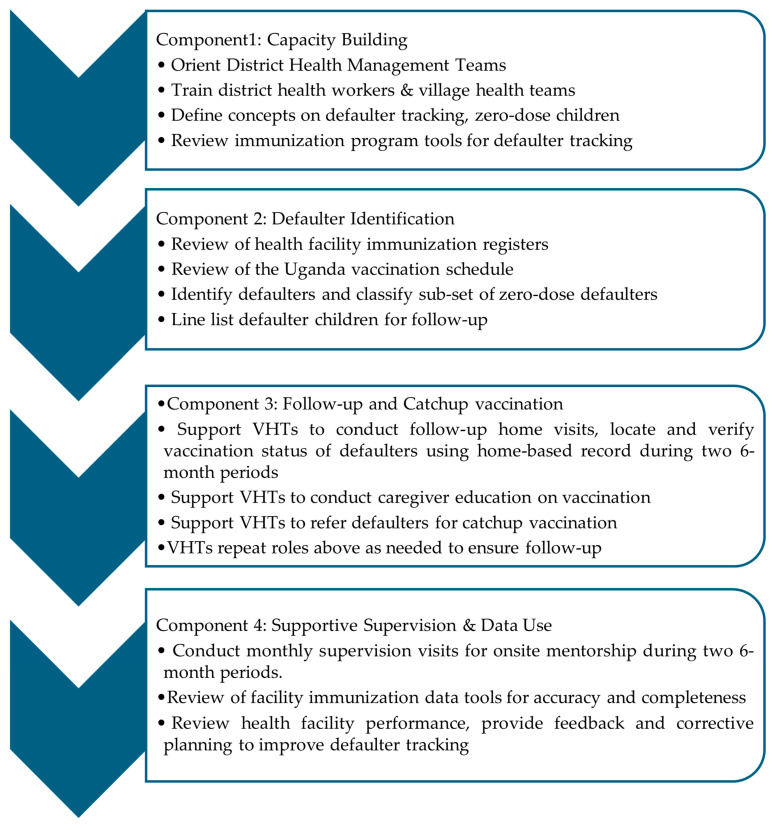
Summary of key components of implementation of the defaulter tracking approach, Uganda 2022 and 2024.

Defaulter tracking targeted children aged 0–59 months who had missed one or more doses of vaccines according to Uganda’s national immunization schedule (defaulters). A defaulter was defined operationally as a child overdue for a scheduled vaccination visit by ≥4 weeks from the return date indicated on the child health card or facility register. This standardized threshold was used to facilitate uniform community tracing across antigen schedules. The defaulter tracking approach was implemented using four sequential components as shown in Figure 1.

First, the District Health Management Teams (DHMTs) and healthcare workers (HCWs) were oriented on identification and follow-up of defaulters, the use of monitoring tools, and on-the-job supportive supervision. The Village Health Teams (VHTs) received orientation on defaulter identification, household tracing, caregiver engagement, documentation, referral procedures, and reporting. The orientation was delivered at district and health facility level by Ministry of Health staff, DHMTs, and Uganda Field Epidemiology Training Program (FETP) personnel as part of implementation rollout. Training content included identification of defaulters from health facility immunization registers, use of defaulter tracking tools, household tracing procedures, caregiver engagement and communication, review of home-based vaccination records, referral for catch-up vaccination, and documentation of outcomes.

Defaulters were identified through systematic monthly review of health facility immunization registers using all antigens in Uganda’s national immunization schedule and catchup guidance at the time of implementation (Appendix A). Because this implementation approach relied on routine health facility immunization records, it primarily identified children with prior contact with vaccination services who had subsequently missed scheduled vaccine doses. Children who had not received DTP1 were considered defaulters if they had prior documented contact with the immunization system (e.g., receipt of birth vaccines) but had failed to return for subsequent scheduled vaccinations. In practice, these children were identifiable in the immunization registers because some had records of birth vaccines (e.g., BCG and oral polio vaccine (OPV-0) at birth) but had not returned for subsequent vaccinations, including DTP1. HCWs compiled line lists in a stand-alone defaulter tracking register (Appendix B). These lists were updated monthly throughout the implementation period and shared with VHTs for community follow-up. During follow-up, VHTs conducted at least one monthly household visit after receiving the defaulter list and made repeat visits, when necessary, until the defaulter received catch-up vaccination or the implementation period ended. At the household visit, the VHTs checked the home-based vaccination record, verified their vaccination, provided caregiver education, and referred the child for catch-up vaccination, if needed.

Supportive supervision and monitoring visits were conducted monthly throughout each of the six-month implementation periods by teams comprising Ministry of Health staff, field epidemiologists from the Uganda FETP, and DHMT members (including the District Health Officer, Assistant District Health Officer, Immunization Focal Person, and district Biostatistician). The supportive supervision included validation of defaulter tracking records at the health facilities and communities, review of immunization data quality, measuring progress and performance of the health facilities, and technical assistance to improve reporting accuracy, completeness, and timeliness.

### 2.3. Monitoring

Routine program monitoring was conducted as part of the supportive supervision and monitoring visits described above. These were conducted monthly to track implementation progress and assess trends in catchup vaccination of defaulters. Monitoring activities emphasized verification of VHT household follow-up, analysis of immunization data trends, and provision of structured feedback to strengthen performance in defaulter tracking.

The supervision teams conducted in-person visits to all vaccination-providing health facilities to review defaulter follow-up activities, examine changes in coverage among previously missed children, and assess the quality of record keeping for the health facility defaulter registers. The teams also reviewed the VHT registers to assess predefined monitoring indicators related to defaulter tracking and follow-up, including vaccines missed and vaccination outcomes after follow-up. In addition, during monthly supervision visits VHTs reported to supervisors the reasons for non-vaccination after follow-up that were obtained from discussion with caregivers during household visits. Along with individual vaccination status of defaulters, caregiver-reported reasons for non-vaccination of a defaulter were recorded in VHT registers and later extracted for analysis. Data from the VHT register were de-identified, entered into Research Electronic Data Capture (REDCap) database [26,27], and then analyzed descriptively using REDCap (version 13.7.20 released on 26 October 2023)) and R (version 4.3.1 released on 16 June 2023) [28]. These data were used to understand the reach of VHTs follow-up in the community, initial vaccination status of identified defaulters and type and number of catchup vaccine doses that defaulters received. Because this manuscript summarizes routinely collected implementation data rather than a comparative effectiveness evaluation, analyses were descriptive and focused on operational implementation outcomes, including identification, tracing, verification, follow-up, and catch-up vaccination among identified defaulters.

## 3. Results

We summarize aggregate implementation experience using routinely compiled programmatic data, and the results are presented at overall implementation level rather than stratified by district or implementation period. Across the four selected districts in Uganda, community-based defaulter tracking activities took place in 102 health facilities and the associated catchment communities. The proportion of total health facilities that implemented defaulter tracking varied by district: Hoima (*n* = 32/32; 100%), Masaka (*n* = 28/28; 100%), Nakasongola (*n* = 24/40; 60%), and Bukomansimbi (*n* = 18/24; 75%). A total of 24 DHMT staff, 202 HCWs and 2048 VHT members and community leaders were trained to identify, follow-up and refer defaulters for catch-up vaccination.

### 3.1. Defaulter Identification

**Figure 2:**
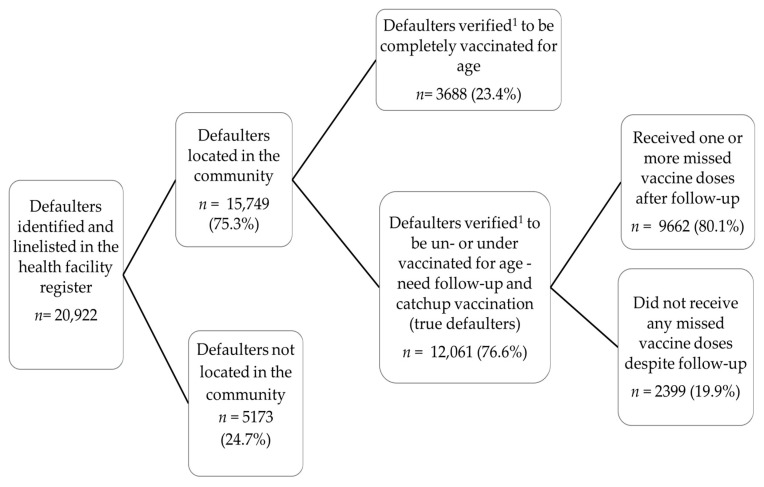
Summary of outcomes of defaulter tracking and catch-up vaccination among 20,922 defaulters in 102 health facilities in Uganda, 2022 and 2024. ^1^ Verified by the VHTs checking defaulters’ homebased vaccination records (child health card).

Across the 102 participating health facilities with a catchment population of 34,069 children aged 0 to 59 months, 61.4% (*n* = 20,922) were identified as defaulters from health facility line listing at the beginning of implementation. VHTs located 15,749 (75.3%) defaulters in the community, among which, 3688 (23.4%) defaulters were found to be previously vaccinated when VHTs checked defaulters’ homebased vaccination records (child health card), leaving 12,061 (76.6%) as true defaulters (Figure 2). All proportions in this section refer to child-level outcomes unless otherwise stated.

### 3.2. Defaulter Vaccines Missed and Tracking Outcomes

Among the 12,061 true defaulters, the most frequently missed vaccine doses were the first dose of measles–rubella (MR) accounting for 11,803 (97.9%) defaulters, followed by DTP3 with 5590 (46.3%). The number of defaulters were fewer for vaccines scheduled in the first 6 weeks of life, with 2649 (22.0%) for DTP1 and 865 (7.2%) for Bacillus Calmette-Guérin (BCG). The second dose of measles–rubella vaccine (MR2) was introduced in October 2022 [29], and thus less children were eligible to receive this vaccine; therefore, the number of MR2 defaulters were fewest in absolute numbers (*n* = 829; 6.9%).

**Figure 3:**
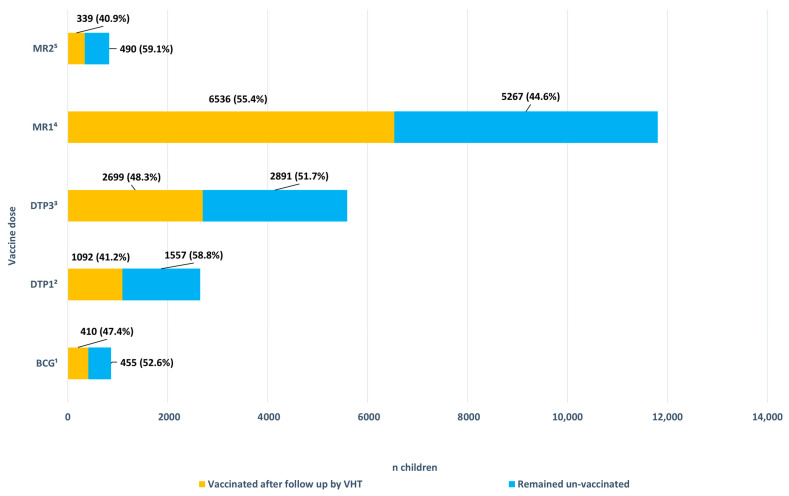
Vaccination status of 12,061 true defaulters located in 4 districts of Uganda, 2022 and 2024. ^1^ BCG: bacillus Calmette–Guérin vaccine; ^2^ DTP1: diphtheria–tetanus–pertussis-containing vaccine, dose 1; ^3^ DTP3: diphtheria–tetanus–pertussis-containing vaccine, dose 3; ^4^ MR1: measles–rubella containing vaccine, dose 1; ^5^ MR2: measles–rubella-containing vaccine, dose 2.

Overall, at the end of the intervention period, 80.1% (*n* = 9662) of true defaulters had received one or more of the vaccines they had missed after follow-up by the VHT. Figure 3 summarizes the vaccination outcomes after VHT follow-up of children verified as true defaulters (*n* = 12,061) through community/household visits to review home-based vaccination records.

The vaccine-specific proportions presented below refer to antigen-specific catch-up vaccination among children who had missed that specific vaccine dose, rather than overall child-level outcomes.

Among all vaccines that initial located defaulters were missing, the highest proportion received MR1 (55.4%, 6536/11,803) followed by DTP3 (48.3%, 2699/5590), BCG (47.4%, 410/865) and DTP1 (41.2%, 1092/2649).

### 3.3. Caregiver Reasons for Non-Vaccination of Their Child Despite Follow-Up by the VHT

For children who did not receive their missed vaccine doses despite repeated follow-up by VHTs (*n* = 2399, caregivers reported various reasons explaining why these defaulters had not received their missed vaccines (Table 1). These reasons were grouped into broad operational categories to better reflect differing implementation challenges as follows:

Population mobility and tracing challenges: Among children not successfully linked to vaccination following follow-up, 49.5% (*n* = 1188) were reported by VHTs as having relocated outside the original community or catchment area, while 8.1% (*n* = 195) were reported as not known at the recorded household location, suggesting possible data quality limitations or incorrect tracing information.

Caregiver delay or competing priorities: Some caregivers indicated they planned to bring the child for vaccination later (16.3%, *n* = 391), while others reported being busy (4.1%, *n* = 99) or facing transportation challenges (1.9%, *n* = 45).

Caregiver refusal or hesitancy-related reasons: Caregiver disinterest in further vaccination was reported for 15.4% (*n* = 370), while smaller proportions cited vaccine safety concerns (1.1%, *n* = 27), religious or cultural beliefs (1.0%, *n* = 24), or belief that the child had already received sufficient vaccines (1.2%, *n* = 29).

Health system-related constraints: Vaccine stockouts were reported in 1.3% (*n* = 31) of cases.

## 4. Discussion

This implementation experience demonstrates the operational feasibility of using community-based defaulter tracking to identify, locate, and support catch-up vaccination among children who missed scheduled routine immunization services in selected districts in Uganda during post-COVID-19 recovery efforts. Approximately three out of four children identified from facility-based defaulter registers were successfully traced in the community, and most verified defaulters received one or more missed vaccine doses following follow-up. However, important operational challenges—including data quality limitations, population mobility, and caregiver-related barriers—affected implementation outcomes and should be considered when interpreting these findings.

Structured collaboration between health facility staff and community members anchored in the routine use of immunization data appears to be an operationally useful approach for supporting catch-up vaccination. In Uganda, regular review of facility-based child health registers led to translation of data into action, through the use of defaulter line lists used by VHTs for targeted follow-up. A high proportion (76%) of listed defaulters were tracked in the community and returned for catch-up vaccination, suggesting the feasibility of VHT-led linkage services in this implementation context. This compares favorably with findings from another Ugandan study that tracked and linked 68% of defaulters to immunization services [24], as well as outcomes from Uganda’s Big Catch Up (BCU) in October 2024 which led to catch-up of 89% of DTP1 defaulters, 70% of DTP3 defaulters and 50% of MR1 defaulters [30]. While direct comparison should be interpreted cautiously due to differences in implementation context and design, these findings are broadly consistent with published evidence suggesting that coordinated community–facility collaboration may support improved vaccination uptake in low- and middle-income countries (LMICs) [31]. Evidence from systematic reviews [32,33], further show that combined community engagement and health provider interventions are more effective than isolated strategies in improving vaccination uptake in LMICs. Additionally, these systematic reviews highlight that community-based vaccination strategies and home-based follow-up can significantly increase immunization uptake. Ensuring sustainability of structured collaboration between health facility staff and VHTs will require adequate resourcing, including operational support (i.e., incentives or stipend for transport reimbursement and communication airtime for tracing activities) and remuneration for VHTs and HCWs [34]. To help support the role of VHTs in immunization, national guidelines should formally define the VHT roles in immunization tracking and follow-up, and DHMT should ensure integration and linkage with other community health services to avoid duplication.

Supportive supervision by DHMT staff appeared to be an important implementation enabler for community-based defaulter tracking. Monthly supportive supervision provided on-the-job mentorship to HCWs and VHTs, strengthening core processes such as routine review of immunization registers, identification and line listing of defaulters, use of tracking tools, and structured follow-up for each child. DHMT staff also fostered motivation among VHTs and healthcare workers, monitored VHT household visits, and reinforced a culture of data use for decision-making which all helped to improve follow-up and linkage of defaulters to services. Our experience aligns with evidence from other published studies showing that supportive supervision and mentorship in LMICs improves health worker performance and service quality, with impact driven more by the quality of engagement, local context, supervisor support, and structured problem-solving approaches than by supervision frequency alone [35,36,37]. However, the relative contribution of supportive supervision to observed implementation outcomes was not directly measured in this study.

The lowest proportion of defaulters was observed for BCG and DTP1 vaccines scheduled in the first six weeks of life, while the largest number of defaulters was observed for DTP3 and MR1 vaccines targeting children 3–9 months of age, suggesting challenges in children completing later vaccine doses, particularly during the transition into the second year of life. Low proportions of defaulters for BCG and DTP1 vaccines suggest that once caregivers engage with immunization services after birth, their willingness to vaccinate remains high. The observed pattern of lower defaulter proportions for early antigens such as BCG and DTP1 compared to later vaccine doses (DTP3, MR1 and MR2) is consistent with national data from Uganda’s 2024 BCU [30]. The high proportion of missed MR1 vaccination suggests gaps in continuity beyond early infant visits, indicating a need for caregiver reminder systems. Additionally, incomplete uptake despite follow-up highlights the importance of integrating defaulter tracking with service delivery, including vaccination during home visits where feasible. In our approach, increasing defaulters for subsequent vaccine doses may be due to lack of awareness and knowledge of the importance of completing the vaccination schedule. Lack of vaccination knowledge among caregivers is a crucial factor influencing childhood vaccination uptake [38]. Increasing caregiver vaccine knowledge using acceptable information, reminders and nudges has been shown to increase childhood vaccine uptake [39]. Similarly, motivational interviewing approaches that engage caregivers in respectful dialog to explore concerns and strengthen confidence in vaccination have also been shown to reduce vaccine hesitancy and improve uptake [40]. Beyond caregiver knowledge, cultural norms that prioritize health-seeking in the immediate postnatal period, with less emphasis on preventive health visits as a child grows older, may also contribute to the declining completion of later vaccine doses as the child grows older. Access barriers, cited by only 1–2% of caregivers, appeared minimal, reflecting the reach of Uganda’s primary health care network. This indicates that missed vaccine doses were more related to utilization of immunization services than outright caregiver refusal to vaccinate their child. Strengthening defaulter tracking for children missing vaccine doses scheduled later in infancy through targeted reminder systems, counseling at earlier visits, and proactive follow-up of children approaching DTP3 and MR1 milestones may help reduce defaulters and improve completion of the immunization schedule.

Migration outside the community was the most frequently reported reason (49.5%) for defaulters not being reached during follow-up. In this case, this referred to internal population movement within Uganda, including movement outside the original health facility catchment area. In the absence of systems linking immunization records across facilities, population movement remains a major barrier to optimal community-based defaulter tracking. Evidence from several studies has shown that migrant children are less likely to be fully immunized than non-migrants [41,42]. This disparity is not solely attributable to mobility itself but also reflects broader structural barriers, including limited continuity of care, administrative exclusion, and reduced trust in health systems, which may further undermine immunization uptake among migrant populations [41]. Children who move across villages or districts often become “lost” to the health system, leading to inaccurate estimates of the target population, inflated defaulter lists, and inefficient follow-up. To address this challenge, digital innovations such as electronic immunization registries or interoperable immunization information systems may improve continuity of follow-up across geographical areas [43,44]. In the interim, VHTs provide important local knowledge to help identify population movement, update facility records, and improve the accuracy of defaulter lists.

Caregiver-related barriers also contributed to incomplete catch-up vaccination. Some caregivers expressed disinterest, delayed acting despite indicating intent, or cited practical constraints such as time, transportation, or vaccine concerns. These findings may reflect key dimensions of vaccine hesitancy—possibly characterized by concerns about vaccine safety and effectiveness, low confidence in health systems, and practical barriers to access—which are increasingly recognized as constraints to optimal childhood vaccination uptake in LMICs [45]. The published literature from Uganda and similar settings suggests that vaccine confidence, knowledge, household decision-making dynamics, and caregiver experience with health services can influence childhood vaccination uptake [46,47,48]. These findings reinforce that even when access barriers are addressed through VHT follow-up and vaccine availability, targeted communication strategies, strong interpersonal communication, trust-building, and continuous community engagement are essential to address underlying hesitancy, vaccine safety concerns and contextual influences on caregiver decision-making about catchup vaccination.

Poor data quality in health facility registers limits the efficiency of defaulter tracking. Nearly one in four identified defaulters in our study were found to be age-appropriately vaccinated at follow-up, pointing to data recording gaps in health facility records as well as a mismatch between health facility registers and home-based vaccination records (HBRs), which is a known challenge in Uganda [49]. However, this finding may also partly reflect the time lag between identification of defaulters from health facility registers and subsequent household visits, during which some children may have received missed vaccinations. In addition, incomplete updating of health facility registers—potentially due to healthcare workers prioritizing documentation in HBRs over facility-based tools—may further contribute to these discrepancies.

Approximately one-quarter of listed defaulters could not be located, largely due to health facility data recording inaccuracies such as misspelled names, incorrect age and sex, outdated address/location information, and missing caregiver contact information. These documentation gaps reflect a weakness in data completeness at the point of primary collection, resulting in poor concurrence between health facility registers and HBRs. Inaccurate or outdated records can result in misclassification of children’s immunization status and inflated defaulter lists, which have implications for program efficiency, diverting limited defaulter-tracking resources away from true defaulters. Similar immunization data gaps have been observed across sub-Saharan Africa, often linked to inaccurate child records, high HCW workloads, inadequate HCW training, and parallel paper-based systems [50]. To improve the effectiveness of defaulter tracking, it is essential to strengthen data quality at the point of collection, including reinforcing HCW and VHT training on accurate documentation, routine data quality audits, and regular harmonization and reconciliation of health facility-based, community-based and HBRs. Such strategies could further reduce misclassification and ensure that defaulter-tracking resources are directed towards actual defaulters.

The strength of the Ugandan experience of defaulter tracking lay in the collaborative and methodical use of defaulter tracking registers within routine district and community health structures. However, there are limitations to be considered when interpreting the outcomes that were achieved. First, monitoring relied heavily on routine health facility records and community health worker reports, which may be incomplete or inaccurate especially for address/location information and vaccines missed. Second, because initial identification relied on facility immunization registers, this approach primarily identified children with prior documented contact with vaccination services and could not comprehensively identify true zero-dose children with no prior health system contact. Third, because this was a descriptive implementation experience in purposively selected districts without a comparison group, findings should not be interpreted as evidence of comparative effectiveness or causal impact. Some children may have received catch-up vaccination even without this implementation. Fourth, the same routine implementation tools were also used for monitoring, creating the possibility of reporting bias, over-reporting, or improvements in documentation influencing observed outcomes. Finally, concurrent health activities, including immunization campaigns and healthcare worker training, may have influenced implementation outcomes independently of the defaulter tracking activities.

## 5. Conclusions

In Uganda’s post-COVID-19 recovery period, data-driven identification and community-led follow-up of defaulters was a feasible and operationally useful approach for supporting catch-up vaccination among identified and located children who had missed scheduled routine immunization services in the selected districts. Improving defaulter tracking will require continued investment in interoperable electronic immunization data systems that enable identification of children and continuity of vaccination across internal geographic boundaries, targeted community sensitization about catch-up vaccination, caregiver reminders, and strong interpersonal communication between VHTs and caregivers, regular supportive supervision, and integration of defaulter tracking into routine immunization service delivery at every health facility. This implementation experience suggests that community-driven defaulter tracking may provide a practical approach for reducing missed opportunities for vaccination and supporting recovery from immunization service disruptions, particularly in settings with established community health structures.

Beyond immunization, the Ugandan experience demonstrates how structured community engagement and iterative data use can strengthen primary health care systems.

## Figures and Tables

**Table 1:** Caregiver-reported reasons why defaulters (*n* = 2399) did not receive missed vaccine doses after follow-up by Village Health Teams, Uganda 2022 and 2024.

Caregiver Reason for Their Child Not Receiving Catch-Up Vaccination ^1^	*n* (%)
Family migrated out of the community	1188 (49.5%)
Caregiver indicated they planned to bring the child for vaccination at a later time	391 (16.3%)
Caregiver did not want to bring the child for vaccination (disinterest)	370 (15.4%)
Lost to follow-up (Child/caregiver not known at the recorded household location **^2^**	195 (8.1%)
Caregiver busy	99 (4.1%)
Transportation challenges	45 (1.9%)
Religious and cultural beliefs	24 (1.0%)
Vaccine stockout	31 (1.3%)
Caregiver had vaccine safety concerns	27 (1.1%)
Caregiver felt the child had enough vaccines and didn’t want them to receive any more	29 (1.2%)

^1^ Among defaulters missing one or more vaccine doses that did not receive catchup vaccines despite follow-up; ^2^ Village Health Teams reached the recorded household location but neighbors or household members reported that the child or caregiver was not known or could not be identified at that location, and therefore was considered as lost to follow-up.

## Data Availability

The data supporting the findings of this study are not publicly available as they contain individual-level health information collected through the routine immunization system. De-identified data may be made available from the corresponding author upon request and with permission from the Ugandan Ministry of Health.

## Abbreviations

The following abbreviations are used in this manuscript:

AFENET	African Field Epidemiology Network
BCG	Bacillus Calmette–Guérin vaccine
BCU	Big Catch Up
CDC	U.S. Centers for Disease Control and Prevention
CHW	Community healthcare worker
DHMT	District health management teams
DTP	Diphtheria–tetanus–pertussis-containing vaccine
EIRs	Electronic Immunization Registries
EMRs	Electronic Medical Records
EPI	Expanded Program on Immunization
FETP	Field Epidemiology Training Program
HBR	Home-based Vaccination Record
HCWs	Healthcare Workers
MR	Measles and Rubella vaccine
RED/REC	Reaching Every District/Reaching Every Child
UNEPI	Uganda National Expanded Program on Immunization
VHT	Village health teams

## Appendix A

Minimum and Maximum age and intervals between multiple doses of the same vaccine for interrupted or delayed routine immunization in Uganda, 2022 and 2024.

**Table A1:** Uganda Defaulter Tracking Guidelines.

Vaccine	Minimum Age at First Dose	Minimum Interval Between Doses 1 and 2	Minimum Interval Between Doses 2 and 3	Minimum Interval Between Doses 3 and 4	Comments
BCG ^a^	Birth				Give at earliest opportunity after birth ^a^
Hepatitis b (birth dose)	Birth				Give at earliest opportunity after birth but not after 6 weeks of age
Oral Polio vaccine (OPV)0	Birth				
OPV	6 weeks	4 weeks	4 weeks		
Pentavalent ^b^	6 weeks	4 weeks	4 weeks		
Pneumococcal Conjugate Vaccine (PCV)	6 weeks	4 weeks	4 weeks		
Rotavirus	6 weeks	4 weeks	4 weeks		Not recommended > 24 months (2 years) of age.
Inactivated Polio Vaccine (IPV)	6 weeks	4 weeks			
Measles—Rubella (MR) ^c^	9 months (6 months, see comments)	4 weeks (and >1 year of age, for 2nd dose)			In certain cases, a supplementary dose of measles vaccine can be given as early as 6 months of age.
Yellow Fever (YF) ^c^	9 months				Life protection

^a^ The bacillus Calmette–Guérin (BCG) vaccine is recommended for unvaccinated Tuberculin Skin Test (TST)- or Interferon-Gamma Release Assay (IGRA)-negative older children, adolescents, and adults from settings with high incidence of tuberculosis (TB) and/or high leprosy burden and those moving from low to high TB incidence/ leprosy burden settings. ^b^ Diphtheria, Tetanus, Pertussis (whole-cell), Hepatitis B, and *Haemophilus influenzae* type b (Hib) Conjugate Vaccine; and ^c^ Live vaccines should be given on the same day as each other or separated by 4 weeks.

## Appendix B

**Table A2:** Defaulter Tacking Register Template/Logbook.

Routine Immunization Defaulter Tracking Template								
District Name ………………………………………… Health Facility ……………………………………………………………. Month ………………………………… Year ……………………								
**S/N**	**Parish**	**Village**	**Parent/Caretaker**	**Child**				**Comment/Date immunized**
				**Reg No**	**Name of child**	**DOB**	**Antigen(s) due**	
1			Name…………………………………………………… Tel No. …………………………………………………					

2			Name ………………………………………………… Tel No. ………………………………………………					

3			Name ………………………………………………… Tel No. ………………………………………………					

4			Name ………………………………………………… Tel No. ………………………………………………					

5			Name ………………………………………………… Tel No. ………………………………………………					

6			Name ………………………………………………… Tel No. ………………………………………………					
